# The Importance of Regulation in Natural Immunity to HIV

**DOI:** 10.3390/vaccines9030271

**Published:** 2021-03-18

**Authors:** Laurence Blondin-Ladrie, Matheus Aranguren, Kim Doyon-Laliberté, Johanne Poudrier, Michel Roger

**Affiliations:** 1 Axe Immunopathologie, Centre de Recherche du Centre Hospitalier de l’Université de Montréal (CRCHUM), Montréal, QC H2X0A9, Canada; laurence.blondin-ladrie@umontreal.ca (L.B.-L.); mat.aranguren@gmail.com (M.A.); K.DOYONLALIBERTE@VIDEOTRON.CA (K.D.-L.); 2 Département de Microbiologie, Infectiologie et Immunologie de l‘Université de Montréal, Montréal, QC H3C3J7, Canada; 3 Institut National de Santé Publique du Québec, Montréal, QC H2P1E2, Canada

**Keywords:** natural immunity, HIV, highly exposed seronegative (HESN), regulatory cells

## Abstract

Worldwide, most Human Immunodeficiency Virus (HIV) infections are acquired through heterosexual intercourse, and in sub-Saharan Africa, 59% of new HIV infections affect women. Vaccines and microbicides hold promise for preventing the acquisition of HIV. To this end, the study of HIV highly exposed seronegative (HESN) female commercial sex workers (CSWs), who constitute a model of natural immunity to HIV, provides an exceptional opportunity to determine important clues for the development of preventive strategies. Studies using both female genital tract (FGT) and peripheral blood samples of HESN CSWs, have allowed identifying distinct features, notably low-inflammatory patterns associated with resistance to infection. How this seemingly regulated response is achieved at the initial site of HIV infection remains unknown. One hypothesis is that populations presenting regulatory profiles contribute to the orchestration of potent anti-viral and low-inflammatory responses at the initial site of HIV transmission. Here, we view to update our knowledge regarding this issue.

## 1. Introduction

Approximately 38 million people are living with HIV/Acquired Immuno-Deficiency Syndrome (AIDS) worldwide. Most HIV infections are acquired through heterosexual intercourse, and in Africa, 59% of new infections affect women [1]. There is currently no cure for HIV and the development of vaccines and microbicides remains the best solution to eradicate the pandemic. We believe that efforts to develop effective devices should aim at mimicking and/or soliciting innate and adaptive immune responses, such as those observed in the context of natural immunity to HIV. Among heavily HIV-exposed female commercial sex workers (CSWs), some women remain uninfected (resistant) despite several years of exposure. These HIV highly exposed seronegative (HESN) individuals therefore constitute an exceptional model of natural immunity to HIV. To this end, the comparison of samples from HESN CSWs and women involved in sex work but not yet HESN should always be considered to control the effects of sex work itself on genital immunology [2]. Several groups have identified HESN individuals among African female CSWs as well as among other CSW cohorts around the world [3,4,5,6]. Analyses of blood and genital samples from HESN CSWs have demonstrated potent anti-inflammatory conditions. We established a cohort of female CSWs in Benin and have identified individuals who remain HIV-uninfected after more than seven years of active prostitution. So far, analysis of the host response in the female genital tract (FGT) and in peripheral blood of Beninese HIV-infected and HESN CSWs allowed us to identify distinct features, notably inflammatory patterns associated with either susceptibility or resistance to infection. More precisely, we have shown that Beninese HESNs maintain low genital inflammatory conditions to prevent HIV infection [7,8], a feature which has also been observed in other groups of HESNs [3,5,6]. In agreement with these observations, we recently reported that B lymphocyte stimulator (BLyS)/B-cell activation factor (BAFF) levels were reduced in both blood [9] and FGT [10] of Beninese HESNs when compared to HIV-uninfected non-CSWs and HIV-infected CSWs. Interestingly, these HESNs presented elevated interferon (IFN)-α levels in their genital samples, which were concomitant with elevated frequencies of myeloid Human Leukocyte Antigen-DR isotype (HLA-DR)^+^ cells expressing high levels of regulatory molecules [11]. How this anti-viral but seemingly regulated response is achieved at the initial site of HIV infection remains unknown. We believe that populations presenting regulatory profiles may contribute to the orchestration of potent anti-viral and low-inflammatory responses at the initial site of HIV transmission.

## 2. The FGT

The complexity of the FGT has been previously thoroughly reviewed [12]. To summarize, the FGT and associated lymphoid structures are part of the mucosal associated lymphoid tissue (MALT), which also includes the gastro-intestinal lymphoid tissue (GALT). As such, studies on the GALT have often been used to guide FGT investigations in different populations due to the difficulty in obtaining ethical consent and samples. A unique particularity of FGT immunity is that it is tightly regulated by a hormonal/inflammatory process throughout the menstrual cycle, having to deal with the pressure of procreation, as well as with commensal and pathogenic microbial control. The FGT is subdivided into phenotypically distinct upper and lower regions, which are separated by a transformation zone, which is highly populated by HIV target cells (see below). The upper FGT is composed of the uterine endometrium, fallopian tubes and the endocervix, which level of sterility depends on the menstrual phase. The upper FGT is covered by single layered columnar epithelial cells, it also presents lymphoid aggregates, which can vary during menstrual cycle and are composed of cluster of differentiation (CD)8+ T-cells surrounding a B-cell core, encapsulated by macrophages. The lower FGT consists of the non-sterile vagina and the ectocervix and is covered by a stratified squamous epithelium. It is in this region that is located the commensal microflora (see below). The first line of defense in the FGT is the acidic milieu and the mucus layer containing immune mediators, such as antimicrobial agents, antibodies (Abs), complement and cytokines, forming a chemical barrier [13]. Abs are pivotal to FGT mucosal immunity. Unlike the GALT where immunoglobulin (Ig)A predominate [14], at FGT mucosal sites, both IgA and IgG are produced, but IgG predominate [14,15]. IgG are produced locally in the lamina propria by local plasma cells or systemically and can be transported across epithelial cells, via the neonatal Fc receptor (FcRn), to the lumen [15]. Moreover, this transport is bidirectional, as FcRn can transport antigen complexed-IgG back from the lumen and thereafter solicit regional effector functions from leukocytes (see section on natural immunity) [14,15]. IgA, and to a lesser extent IgM, are produced locally, mainly as polymeric containing a joining J chain. Both can translocate across epithelial cells to generate luminal secretory IgA (SIgA) or IgM (SIgM) via the polymeric Ig receptor (pIgR). Mucosal SIgA, and to some extent SIgM, can mediate protection by trapping, neutralizing and preventing transcytosis [14]. Moreover, in GALT, antigen complexed SIgA can be taken from the apical to basolateral side of the epithelium through M cells, and thereafter mediate leukocyte effector functions (see section on natural immunity) [14]. It is most likely that populations guarantying the function of M cells are present in the FGT, but have yet to be characterized.

Epithelial cells are pivotal to mucosal integrity and form an uninterrupted barrier between the lumen and underlying cells. Through cross-talk with sub-mucosal dendritic cells (DCs) and Langherans cells (LCs), epithelial cells contribute to the orchestration of innate and adaptive mucosal responses and maintenance of a homeostatic balance between tolerance vs defense [12,16,17,18]. As such, pattern recognition receptor (PRR) sensing, such as via Toll-like Receptors (TLRs), contributes to the production of chemokines and cytokines that help shape the outcome of innate and adaptive mucosal responses [12,16,17,18].

### 2.1. The FGT in the Context of HIV

The number of sexual partners and their risk characteristics as well as early sexual debut (before age 14) and the failure to use condoms are among the best documented behavioral risk factors for HIV sexual transmission [19]. Intravaginal practices such as cleansing or usage of products for hygienic or sexual reasons can also lead to increased HIV infection risk [20,21]. Among the most compelling biologic risk factors are the presence of vaginosis and sexually transmitted infections (STIs), high viral load and low CD4 lymphocyte counts in the infectious contact, and possibly viral virulence and tropism [19]. HIV can cross the epithelial barrier when the junctions between the epithelial cells are loosened due to pro-inflammatory factors such as tumor necrosis factor (TNF) [22] and/or by sustained activity of effector T-cells in the early stages of the infection [23]. Also, epithelial cells, which appear to be not infected, allow for endocytosis of the virus via the galactosylceramide receptor (GalCer), which is highly expressed at the luminal pole of epithelial cells, and can bind to the HIV Envelope (Env) glycoproteins gp120 and gp41 [24] permitting the transfer of the virus to sub-mucosal DC populations and transmission to CD4^+^ target T-cells [25,26,27]. Another way for endocytosis of HIV by FGT epithelial cells is via gp340, a scavenger receptor, activating TLR7 signaling then promoting the production of pro-inflammatory thymic stromal lymphopoietin (TSLP), activating sub-mucosal DCs and promoting HIV transmission to CD4^+^ target T-cells [28].

Several reports in humans and non-human primates (NHP) have suggested that LCs and DCs at mucosal surfaces are of the earliest leukocytes to be exposed, and possibly infected by HIV or Simian Immunodeficiency Virus (SIV), and migrate to lamina propria and draining lymph nodes to facilitate transmission of the virus to permissive cells [16,27,29,30,31,32]. The virus can be internalized by mucosal LCs and DCs via C-type lectin receptors such as Langerin or Dendritic Cell-Specific Intercellular adhesion molecule-3-Grabbing Non-integrin (DC-SIGN) (CD209), which are highly expressed by these populations and can bind to HIV Env [33,34,35,36]. After internalization, the virus can be transferred to CD4^+^ C-C Motif Chemokine Receptor (CCR)5^+^ effector target T-cells either locally or in lymphoid organs [32]. LCs can actually cluster with DCs to favor cross-presentation of HIV to CD8^+^ T-cells [33]. The HIV vaginal infection model was mostly developed by studying SIV infection in NHP [37,38]. In this model, it was shown that following vaginal transmission, the preferential target cells were CD4^+^ T-cells of the T helper (Th)17 lineage [39,40,41]. Strong doses of SIV in the vaginal mucosa were shown to cause an increase of the chemokine macrophage inflammatory protein-3 alpha (MIP-3α)/Chemokine C-C motif ligand 20 (CCL20), produced by both murine [42] and human [43] mucosal epithelial cells, and which attracted populations such as plasmacytoid DC (pDC), Th17 and LC precursors at the cervical epithelium [38,43]. The principal ligand of CCL20 being CCR6, which is expressed by immature LC/DC, pDC and Th17 cells [44]. Studies demonstrated that early blocking of CCL20 prevented cellular recruitment and establishment of an inflammatory milieu, reducing infection despite repeated exposures to SIV [45,46].

It is now widely assumed that mucosal Th17 effectors are the main targets for HIV/SIV and massive depletion of these cells following infection establishment results in an eventual critical loss of balance favoring accumulation of cells with a T-cell regulatory (Treg) phenotype in HIV/SIV infected subjects [47,48,49]. In infectious or inflammatory conditions, certain DC populations produce the enzyme indoleamine 2,3-dioxygenase (IDO), which is up-regulated by IFN-α and TLR agonists [42,50]. This enzyme is key in the regulation of the balance between Treg/Th17 [51]. Augmentation of IDO causes an imbalance in the ratio between Treg/Th17 marked by a depletion of Th17 and a gradual expansion of Tregs [19,23,38,40,46,52]. This imbalance contributes to a persistent inflammatory state in the context of HIV chronic infection [49]. Moreover, this dysregulation is increased by altered CCR6^+^ cell homing patterns [53]. In humans, Th17 differentiation requires transcriptional regulator retinoic acid-related orphan receptor (ROR)ɣt and differentiation factors such as transforming growth factor (TGF)-β, interleukin (IL)-1, IL-6, IL-21 and IL-23 [54,55,56]. Th17 cells produce IL-17, IL-21 and IL-22 to maintain the integrity of the epithelial barrier and defend the organism against bacterial and fungal pathogens [40]. Th17 express α4β7 integrins at their surface to be able to interact with mucosal addressin cell adhesion molecule-1 (MAdCAM-1), which mediates their homing to the gastrointestinal, respiratory and urogenital mucosa [57,58,59,60]. The α4β7 integrin can also be used by certain strains of HIV to facilitate attachment. Although Th17 are the main targets of HIV, it has been shown that mucosa-resident LCs and DCs contribute to the selection process of the HIV founder strain [61,62]. Moreover, in a recent study, macrophages were shown to play an important role in dissemination and cell-to-cell transmission of the virus [63].

Overall, epithelial cells and sub-mucosal DCs and LCs, as well as macrophages, play critical roles in the establishment of HIV infection mainly through PRR sensing, including TLRs which expression and responsiveness are increased in the HIV context [64], and subsequent transmission to Th17 targets. Perturbed activities of these populations nourish the inflammatory context eventually leading to a breach in the Treg/Th17 homeostatic balance in the context of disease progression (reviewed in [16,19]). Of note, in healthy women, neutrophils constitute 10–20% of genital leukocytes. It has been recently shown that stimulation of these neutrophils with HIV-like particles induced the expansion of neutrophil extracellular traps (NETs). Furthermore, pre-formed genital NETs totally inactivated infectious HIV, suggesting an important role for neutrophil responses in the battle against HIV [65].

### 2.2. The FGT Microbiota in the HIV Context

In the past decade, the vaginal microbiome has emerged as a critical modulator of inflammation in the FGT and impacts on susceptibility versus protection against infections.

An indicator of a healthy microflora is characterized by a dominance of the genus *Lactobacillus* spp., which produces lactic acid rendering the environment acidic [66]. Another way that lactobacilli protect the FGT against pathogens is by producing bacteriostatic compounds and antimicrobial peptides [67,68]. Their presence limits the colonization of the FGT by other microorganisms who have to compete for attachment to the epithelium [69].

Vaginal microbiota dysbiosis happens when the dominance of *Lactobacillus* spp. decreases [70]. This shift is marked by an increase in microbial diversity, which involves obligate and facultative anaerobes and more Gram-negative bacteria inducing bacterial vaginosis (BV) [70,71]. This dysbiosis is characterized by a decrease in production of lactic acid resulting in basification of the FGT (pH > 4.5). Another consequence of BV is an elevated level of mucin-degradative enzymes that watery the mucus layer [72]. Due to all of these changes, BV causes disruptions of the mucosal epithelium barrier, increases the inflammatory response and immune activation [73]. Altogether, BV has been reported to increase the risk of acquisition of HIV by approximately 1.5 fold [74]. Multiple studies tend to confirm the conclusions that BV increase inflammatory responses and recruitment of target CD4^+^ T-cells [75,76,77,78]. It has also been shown that BV can augment the risk of acquisition of STIs such as *Chlamydia trachomatis* and *Neisseria gonorrhoeae* [79]. Due to BV persistence [80] and recurrence even when treated [81] this condition can contribute to the population attributable risk (PAR) of HIV infection. In addition, two studies suggest that BV contributes even more to HIV PAR than any other genital condition, except Herpes simplex virus-2 (HSV-2), to HIV susceptibility [82,83]. Infection by the Human Papilloma Virus (HPV) causes activation of cervical CD4^+^ T-cells and a higher recruitment rate of T-cells to the FGT. The high levels of HIV target cells as well as lesions in the epithelium caused by the multiplication of HPV increase the susceptibility to HIV infection [84].

One of the major consequences of STIs is often the destruction of the mucosal barrier by inducing inflammation, damages to the epithelium, and massive recruitment of HIV target cells at the site of infection [85]. Even though they cause such trouble, some STIs, such as *Chlamydia trachomatis* and *Neisseria gonorrhoeae*, are difficult to diagnose because of the lack of symptoms in infected women. Studies have reported that there is a greater than three-fold higher risk of contracting HIV when the person already has an STI [86].

Recently, research on the gut and other niches’ microbiota have described the virome’s role in the microflora. Despite all the studies, the impact of the virome on the FGT remains unclear and must be studied further. Still it can be linked to bacterial variations and other health issues. More studies on this matter could help better understand the interactions in the microflora that can shape the immunity of the FGT [87].

Overall, vaginal dysbiosis and inflammation increase the susceptibility of infection to HIV.

## 3. Natural Immunity to HIV Infection

Natural immunity to HIV infection is likely to be a multifactorial complex process, which involves environmentally and/or genetically determined factors [3,4,5,6]. It is now well accepted that protection against HIV infection in HESN female CSWs is linked with a low-inflammatory/activation profile both in blood and genital compartments [3,5,6]. It was shown that neutralizing proteins, such as anti-proteases and high levels of anti-inflammatory factors are found in the genital mucosa of HESN CSWs [5,88]. Moreover, genetic polymorphisms such as for the interferon regulatory factor (IRF)-1 have been shown to confer a lower infection risk by HIV [89,90]. Still, further exploration is required in order to fully harness elements of natural immunity, which will lead the path to designing preventive strategies. We and others have previously reviewed this concept (reviewed in [3,4,5,6,16,19]) and will therefore herein concentrate our efforts by presenting new aspects of natural immunity against HIV infection such as found in HESN female CSWs, and more particularly as regarding its regulation.

Although it is tempting to attribute a role for differential microbiota composition in the shaping of natural immunity to HIV of HESN individuals, current observations do not point to this fact (see Fowke, Broliden, and collaborators in the *Vaccines* 2021 Special Issue).

To allow procreation, the FGT must become tolerant to seminal fluids but this is accompanied by an increased risk of STIs. To reduce this risk, surveillance from the immune system is increased by recruitment of populations such as DCs and granulocytes as well as changes in the structure of epithelial surfaces [91]. However, the semen contains mediators, such as high levels of IL-10, TGF-β and Prostaglandin E2, inducing a more tolerogenic profile in the FGT [92,93]. In a cohort of CSWs from Puerto Rico, it was found that high exposure to seminal fluids may up-regulate type I IFNε expression in the FGT of HESNs inducing changes that reduce HIV susceptibility, which might explain, in part, the HESN status in this cohort [94].

In a Spanish cohort of serodiscordant couples, it was observed that HESNs produce antibodies that can reduce the binding between HIV gp120 and host receptors α4β7 and DC-SIGN, possibly reducing HIV spreading to the MALT [95].

In a cohort of heavily exposed women from Kenya, HIV-specific CD8^+^ cytotoxic T lymphocyte (CTL) and CD4^+^ T-cell poly-functional responses were found in HESN CSWs [96,97]. When compared to HIV-infected CSWs, CD4^+^ T-cells from these HESNs had a greater ability to proliferate in response to HIV p24 peptides [97,98]. Furthermore, when whole blood of HESNs and HIV-uninfected CSWs from this cohort were compared on a molecular basis, it was found that HESNs have lower expression levels of genes involved in T-cell receptor signaling and host factors required for HIV replication [99]. Moreover, in these same group of women as well as in those from Côte d’Ivoire, HESNs presented HIV Env-specific cross-clade neutralizing mucosal IgA, which blocked viral transcytosis through tight epithelial barriers [3,100,101]. We could not detect substantial IgA1 or IgA2 reactivity to HIV Env glycoproteins in the cervico-vaginal lavage (CVL)s of Beninese HESN CSWs [10]. To date, studies have reported contradictory results regarding anti-HIV-specific IgA responses in the genital tract of HESNs [3,10,100,101,102,103,104,105]. These discrepancies may be linked to the relatively small sample size and/or the different techniques used to detect Env-reactive Abs. Also, the fact that most genital Igs are found in the mucus [106], may preclude the detection of certain Ig isotypes in CVLs. Yet, we have previously detected anti-HIV Env-specific IgG with neutralizing and antibody-dependent cellular cytotoxicity (ADCC) functions in the blood and CVL samples from Beninese HIV-infected CSWs but not in those from HESNs [107]. However, we could detect IgG1 reactivity to gp41 in some HESNs [10], which could be derived from a microbiota reactive, possibly first-line B-cell pool [108], as most gp41 Abs are known to cross-react with microbiota [109]. These observations may imply that natural immunity to HIV in Beninese HESN CSWs is not mediated by IgG neutralizing or ADCC responses, and may involve other functions of Abs that can confer some level of protection, as is now being suggested by a growing body of evidence [110,111,112].

Analyses of correlates of protection from the RV144 vaccine trial had suggested that decreased HIV acquisition was linked to blood derived IgG1 and IgG3-mediated ADCC activity toward the HIV Env V1V2 region [113]. It was shown that RV144 vaccinees bearing certain HLA class II alleles such as DQB1*06 presented increased risk of HIV acquisition, and this was possibly linked with elevated Env-specific IgA that interfered with ADCC activity. However, in RV144 vaccinees who did not bear predisposing HLA alleles, non-neutralizing Env-reactive IgA derived from blood memory B-cells blocked in vitro HIV Env binding to GalCer and mediated in vitro phagocytosis by monocytes. This suggests that the RV144 regimen may have induced a certain level of non-neutralizing IgA in some individuals, which conferred some level of protection [108]. Interestingly, this vaccination regimen was found to elicit more robust responses, notably IgG antibody-dependent cell phagocytosis (ADCP), in vaccinees from South-Africa [114]. The Env-reactivity and functional capacities of IgA were however not reported for the South African vaccinees. Unfortunately, mucosal samples were not collected during the RV144 trial and the reactivity of mucosal Igs have not been assessed. Nevertheless, lessons from the RV144 trial teach us that it is mandatory that we further the exploration of different Ig mediated effector capacities, especially those of ADCP, in HESN CSWs.

Passive immunization studies aiming at the prevention of HIV transmission are promising and have been recently extensively reviewed [115,116]. Administration of broadly neutralizing antibodies (bnAbs) has been shown to protect against HIV acquisition in some clinical trials, one of the most promising being the human monoclonal antibody (mAb) VRC-HIVMAB060-00-AB (VRC01). This bnAb vaccine is under clinical trial for preventing HIV infection in two groups, one being sexually active heterosexual adult women in sub-Saharan Africa and another being men or transgender people having sex with men in the USA, Brazil, and Peru. This study aims to determine minimal concentrations required for passive immunization through bnAbs at mucosal sites. As such, previous passive immunization studies aiming to assess the importance of mucosal Igs in the prevention of HIV transmission revealed them as important weapons [117].

In 2011, the group of Morgan Bomsel had elegantly shown that vaccination of NHP with HIV gp41 virosomes induced mucosal IgA and IgG, which blocked transcytosis and mediated ADCC, respectively, preventing systemic invasion following vaginal simian-HIV (SHIV) challenge [118]. Importantly, these animals lacked serum neutralizing antibody activity, highlighting the importance of effector antibodies at the mucosal portal of entry. A more recent study by this group has shown that gp41 specific IgA can induce ADCP by monocytes and neutrophils [119]. In another study, with NHP, the use of adjuvanted liposomes promoted ADCP mucosal responses by monocytes and neutrophils that protected from SHIV challenge [120]. Along the same lines, refined passive immunization studies revealed full protection of rhesus macaques against SHIV challenge following administration of anti-HIV-1 neutralizing mucosal dIgA2 together with systemic IgG1 with the same epitope specificity [121,122]. Furthermore, mucosal dIgA1 was significantly more protective than dIgA2, highlighting the importance of characterizing different isotypes of IgA, as they differ predominantly in the hinge region and may confer varying effector functions [117]. The fact that these neutralizing Abs were poorly efficient when used alone implies that this passive immunization regimen requires first-line mucosal IgA in conjunction with systemic IgG to prevent virus acquisition.

## 4. Immunoregulatory Populations in the Context of Natural Immunity to HIV

### 4.1. Tolerogeneic DCs

As has been described above, DC are known to be major players in mastering the orchestration of immune responsiveness. We have previously identified endocervical myeloid HLA-DR^+^ cells in the FGT of Beninese CSWs, which in HESNs expressed higher levels of IFN-α, TLR7 and immunoregulatory markers IL-10 and HLA-G when compared to both HIV-infected CSWs and HIV-uninfected non-CSWs [11]. Furthermore, we revealed a myeloid CD103^+^CD14^+^CD11c^+^ population in the FGT that increased in HESNs, and that expressed higher levels of IFN-α and IL-10. Interestingly, the majority of these myeloid CD103^+^CD14^+^CD11c^+^ IFN-α^+^IL-10^+^cells co-expressed HLA-G and Immunoglobulin-like transcript (ILT)-4. Importantly, this profile is reminiscent of tolerogenic DC-10 [123], which secrete high amounts of IL-10, express high levels of HLA-G and ILT-4 and can induce type 1 regulatory cells (Tr1) (CD49b^+^ Lymphocyte activation gene 3 (LAG-3^+^)) via an IL-10-dependent ILT4/HLA-G pathway [124]. Both IFNα and IL-10 are involved in differentiation of Tr1 [125,126] and in vitro studies have demonstrated that monocyte-derived DCs (MoDCs) treated with IL-10 and/or IFNα were rendered “tolerogenic” and up-regulated the inhibitory receptors ILT-3 and ILT-4, which promoted their capacity to induce Tr1 [127,128]. Furthermore, IL-10 is one of the key cytokines inducing HLA-G expression on myeloid cells [129]. The engagement of the inhibitory molecules ILT-2, ILT-3 and ILT-4 on myeloid cells by HLA-G prevents the up-regulation of costimulatory molecules, inhibits maturation and allows them to promote the differentiation of regulatory T-cells [130,131,132]. The fact that myeloid HLA-DR^+^CD14^+^CD11c^+^ cells found in the FGT of Beninese HESNs also expressed high levels of CD103, suggests they could play a similar role to CD103^+^ DCs found throughout the gut lamina propria and thought to be most effective at promoting T regulatory responses and to play a central role in maintaining tolerance and tissue homeostasis [133].

The “DC-10-like” population found to be increased in the FGT of Beninese HESNs is likely to be derived from monocytes, and influenced by the low-inflammatory milieu (reviewed in [134]). For example, in the gut, most DCs are monocyte-derived and are involved in maintenance of homeostasis [133], and it is likely that this also occurs in the FGT as most DCs were shown to express CD14 [135]. In human, monocytes are categorized in three main populations based on their expression levels of CD14 and/or CD16, each representing a stage of differentiation, or more so transition as CD14 goes down and CD16 goes up. They are the predominant CD14^+^CD16^-^ classic, CD14^+^CD16^+^ intermediate and CD14^low/dim^CD16^++^ non-classic populations. Importantly, frequencies of non-classic monocytes are increased in blood in the context of several infectious diseases including HIV infection [134]. As such, non-classic monocytes are known to patrol and sense nucleic acids and viruses via TLR7 and TLR8 receptors [136]. Alterations in the classic vs. non-classic monocyte ratios during pathological conditions may dramatically influence MoDC-mediated immunity. In human, both classic and non-classic monocytes differentiate into MoDC in vitro and present a differential transcriptomic signature. As MoDC derived from non-classic monocytes express CD103, RALDH2 and TCF4 typical of mucosal DC and are thought to play a role in mucosal homeostasis [137]. Moreover, non-classic monocytes preferentially acquired DC features in a model of transendothelial migration [138]. As to whether the relative frequencies of blood monocyte populations are perturbed in HESNs, and whether a particular population is responsible for the generation of the “DC-10-like” population found in the FGT remains to be established.

### 4.2. Tregs and Tr1

Concomitant to the elevated frequencies of tolerogeneic or DC10-like population in their FGT, Beninese HESN CSWs presented elevated frequencies of endocervical regulatory CD4^+^ Tregs [11]. Moreover, these endocervical Tregs expressed higher intensity of programmed cell death protein (PD)-1, as well as IL-10 and cytotoxic T-lymphocyte-associated protein 4 (CTLA4), and this was also found for Tr1 cells [11]. Although this could reflect T-cell exhaustion, we believe that these cells are in an active state [139,140], as reflected by their higher levels of IL-10 expression. These findings are in agreement with a previous study showing elevated frequencies of Tregs in the blood of HESNs from Kenya [141], a finding which has been recently extended (see Fowke, Broliden, and collaborators in the *Vaccines* 2021 Special Issue). The increase in tolerogeneic DC-10-like and Treg populations likely confers an advantage to HESN individuals in preventing the establishment of HIV infection.

### 4.3. Importance of NR4As (Nuclear Receptors 4A)

Orphan nuclear receptor (NR)4A1 (Nur77), NR4A2 (Nurr1) and NR4A3 (NOR1) are transcriptional regulators of differentiation, proliferation, and apoptosis genes. They are important modulators of inflammatory responses and are necessary for monocyte differentiation and Treg development (reviewed in [142]). Importantly, studies of mouse Ly6C^low^ monocytes, which are considered to be the murine analogs of human non-classic monocytes, whereas Ly6C^hi^ correspond to classic monocytes, demonstrated that the transition from Ly6C^hi^ to Ly6C^low^ monocytes depends on NR4A1 expression [143], which is necessary for the generation and survival of Ly6C^low^ monocytes, which are being reduced by 90% in NR4A1-deficient mice [144]. Also, it has been shown that increased NR4A1 expression levels lead to diminished MoDC and T-cell activation [145]. Furthermore, recent studies with NR4A3-deficient mice show that NR4A3 expression is required to skew monocyte differentiation toward MoDCs and allows the acquisition of migratory characteristics required for MoDC function [146].

Since expression of NR4As is necessary for the maintenance of forkhead box P3 (FoxP3) expression and thereby essential for the development of Tregs, there have been reports of several studies where those genes are targeted to modulate Treg responses in cancer [147,148]. Interestingly, when the Tregs that infiltrate the tissue tumor are treated with camptothecin and a cyclooxygenase-2 inhibitor, the transcriptional activity and the induction of those genes are inhibited [148]. This phenomenon is beneficial in mice by breaking the immune tolerance mediated by Tregs that protect the tumor against cytotoxic CD8^+^ CTL cells. These observations help illustrate the potential of NR4A modulation. Thus, it could be possible to hypothesize that high levels of expression of NR4As in immune cells in the FTG of HESN women could help in the maintenance of their low-inflammatory environment, shown to be an important factor in natural immunity against HIV. NR4A receptors are expressed early in response to physiological and pathological stimuli including inflammation. There is growing evidence suggesting that expression levels of NR4As are affected in the context of autoimmunity and infectious diseases. Synthetic regulation of NR4A expression is currently used for treating patients with certain leukemia/lymphomas, and could be envisaged for immunomodulatory purposes.

### 4.4. Innate B-Cells with Breg Potential

In the Kenyan CSWs cohort, late seroconversion occurred in some HESN CSWs and involved waning of responses due to reduced sexual activity and antigenic exposure [149]. These observations could be suggesting that natural immunity may involve innate type “antigen experienced” cell populations that require frequent re-exposure in order to be maintained in a given cellular niche. In this view, HIV Env gp120 binding innate CD1c^+^ B-cell populations were found in the FGT of Beninese HESNs [10], but as to whether they can confer some level of protection remains to be established. A subset of CD1c^+^ B-cells, known as marginal zone (MZ) B-cells, are involved in the early first-line defense against viruses [150]. In humans, MZ B-cells recirculate and have been found in front-line areas such as the sub epithelial lamina propria of the MALT [150]. By trafficking antigen, they promote Germinal Center (GC) reactions, which are essential for the development of effector and memory B-cells [151]. MZ B-cells can also be activated by T-independent signals from neutrophils, DC and/or MoDC, which can lead to class switch recombination (CSR) from IgM toward IgG or IgA [150]. The antibodies produced by these cells are often of low affinity and polyreactive; however, it has been described that they can bind to the HIV Env gp120 glycoprotein through surface lectins and/or polyreactive B-cell receptor (BCR) [152]. We recently showed that MZ and precursor MZ B-cells from the blood of healthy individuals express high levels of CD83 and nuclear receptors NR4A1, 2, 3 [153], which as mentioned above are important regulators of the inflammatory response. Importantly, precursor MZ B-cells demonstrated regulatory functions (Breg) and controlled T-cell proliferation following in vitro activation, and this was dependent on the immunoregulatory surface molecule CD83 [153]. As such, CD83 expression is directly modulated by NR4A1, 2, 3 transcription factors [154]. We thus identified these molecules as potential Breg markers of precursor MZ B-cells, along with CD39/CD73 ectonucleotidases [153]. This is of interest, as yet potent Breg attributes mainly point to IL-10 production. However, in the HIV context, most B-cell populations are driven to over-express IL-10 [155]. As to whether the HIV Env gp120 binding innate CD1c^+^ B-cell population we found in the FGT of Beninese HESNs [10] possess Breg functions and contribute to the low-inflammatory genital profile described for HESNs is attractive but remains to be assessed.

MZ B-cells require BAFF for their selection, expansion, activation and T-independent CSR [150]. BAFF is found as both membrane and soluble forms, the main sources of BAFF being neutrophils, DC and Mo-DC, and to a lesser extent activated T-cells [156,157]. Excess of both membrane and soluble BAFF in the blood of Beninese HIV-infected CSWs has been associated with a dysregulation of the B-cell compartment [9]. Interestingly, Beninese HESNs had lower soluble BAFF levels in their blood and CVLs than HIV-infected CSW or HIV-uninfected non-CSW [9,10]. Also, the relative frequencies of BAFF expressing cells in the genital mucosa of these HESNs were smaller than those observed in the other groups [10]. These raised the possibility that the low levels of soluble BAFF measured in blood [9] and CVLs [10] of Beninese HESNs may be linked to the signals leading to BAFF release. As to whether these are related to advantageous genetic polymorphisms remains to be established. We have analyzed BAFF promoter -871, -2841 and -2701 mutations associated with elevated BAFF plasma levels and susceptibility to auto-immune diseases such as Systemic Lupus Erythematosus and hepatitis C associated cryoglobulinemia [158,159,160] in our Benin cohort and found no association. Sequencing the entire BAFF gene need thus to be envisaged. The lower BAFF levels found in Beninese HESNs did not alter the frequencies of precursor MZ B-cells in blood as they remained similar to those observed in HIV-uninfected individuals [9]. However, we did find lower frequencies of mature MZ B-cells in the blood of HESNs when compared to HIV-uninfected individuals, and this was also found for HIV-infected CSWs [9], which could be suggestive of an active peripheral recruitment and/or exhaustion. Implying that these populations are important in the fight against the virus.

Since the integrity of B-cells, and especially MZ, is closely linked to BAFF, the maintenance of low levels of BAFF in the vaginal mucosa of HESNs can be a key factor of natural immunity.

MZ B-cells are easily activated; it is, therefore, important for them to be controlled or they can lead to autoimmunity [161]. Importantly, it was previously described that invariant natural killer T-cells (iNKT) have this potential [162]. These cells are quite efficient in the modulation of a majority of immune cells such as DC, Natural Killer (NK), T-cells and B-cells [163]. Interestingly, iNKT cells can be activated through glycolipid presentation, which can be mediated by MZ B-cells via CD1c [164]. Based on these observations, it is possible to think that iNKT and MZ-like B-cells play an important role in the maintenance of low-inflammatory conditions in the FGT of HESNs and it is important to study them more carefully in the context of HIV infection and natural immunity.

## 5. Natural Killer Cells

NK cells were shown to play an important role in natural immunity against HIV. NK cells are lymphoid cells that can rapidly respond against infected or transformed cells via potent cytotoxic activity [165]. This activity is mediated by a mix of inhibitory signals and stimulating signals, which are both transmitted by the Killer Immunoglobulin-like Receptors (KIR), which are cell surface receptors that bind to certain HLA molecules [165]. However, NK cells are also strategically placed in the immune response, allowing bridging of both innate and adaptive immunities. Indeed, by producing key cytokines and activating cells such as DCs, NK cells can help modulate the immune response on the infection site [165].

There are two main subsets of human NK cells: the CD56^bright^CD16^low^ subset, which composes up to 10% of all NK cells and is mostly a proliferative subset specialized in cytokine production, and the CD56^dim^CD16^+^ subset, which composes the other 90% and is responsible for the cytotoxic activity of NK cells [165]. HIV infection promotes an expansion of both a CD56^dim^CD16- and a CD56^−^CD16^+^ population that are highly dysfunctional, with lower cytotoxicity potential due to the changes of expression of certain NK receptors and with lower cytokine production [166,167]. It was recently shown that NK cells from the blood of HIV-infected women, most notably CD56^dim^ and CD56^−^ subsets, overexpress the T-cell Immunoreceptor with Ig and ITIM domains (TIGIT) when compared to HIV-uninfected women [168]. Interestingly, TIGIT expression by NK cells correlated with a gain of function, as the TIGIT^+^ subsets expressed higher levels of certain markers associated with a more mature phenotype as well as a stronger cytotoxic activity when compared to the TIGIT^−^ subsets [168]. TIGIT is an exhaustion marker found in T-cells and NK cells in the context of certain malignancies and chronic infections [169,170]. TIGIT expression by memory T-cells correlated with HIV disease progression, with T-cells from elite controllers expressing lower levels of TIGIT than viremic individuals or even ART-treated individuals [171], and a blockage of TIGIT expression by NK cells increased their anti-tumoral cytotoxicity by preventing cell exhaustion [170]. In Beninese HESN women, a slightly higher expression of TIGIT was found when compared to healthy donors [172]. As to whether this slight increase of TIGIT correlates with a gain of function remains to be established.

Several correlations were established between certain alleles of KIR genes and a better control of HIV infection. For instance, a group of HESN individuals from the Montreal Primary HIV Infection (PHI) cohort were shown to possess a higher frequency of KIR3DS1 homozygous individuals when compared to a group of HIV-infected individuals [173]. Moreover, they were found to possess a smaller frequency of other KIR alleles such as KIR2DS4*001, found to correlate with HIV transmission in serodiscordant couples [173,174]. Furthermore, certain HLA/KIR genotype combinations have been associated with a control of viral infection, such as the KIR3DL1*h/*y and HLA-B*57 being more frequently found in HESN than HIV-infected individuals [175]. HLA-B*57 is an HLA-B allele that possesses a mutation encoding for an isoleucine at position 80 of the protein and allows for HIV infection control through the generation of T-cells that respond better to viral epitopes during thymic selection. Furthermore, possession of both HLA-B*57 and KIR3DS1 alleles led to a stronger HIV protection when compared to possessing only one or the other, possibly by allowing for the generation (or “licensing”) of a polyfunctional NK cell subset that highly express IFN-γ, TNF-α and CD107a and thus could contribute to viral control [176,177,178].

While the HLA-B*57 allele is associated with a better control of HIV infection, its presence is equally associated with higher activation levels of CD56^bright^ CD16^low^ NK cells and non-classical CD14^low/dim^CD16^++^ monocytes by bacterial TLR agonists [179]. Indeed, these cells were found to produce higher levels of proinflammatory molecules such as IFN-γ, IL-1β and IL-6 in the presence of Pathogen-associated molecular patterns (PAMPs) such as *Escherichia coli*’s lipopolysaccharide (LPS) when compared to donors possessing other HLA alleles such as HLA-B*44, in vitro [179]. HLA-B*57 is found to correlate with a higher risk of sepsis-related death in HIV-infected and treated individuals [180]. Of importance, IL-6 correlates with a more severe clinical progression in several diseases, including HIV infection [181]. Indeed, IL-1β enriched monocytes overexpress IL-6, which correlates with a higher mortality risk in HIV-infected and treated individuals [182]. Furthermore, IL-6 excess is a prognostic of sepsis, and it is also related to the “cytokine storm syndrome” in several diseases and could lead to autoimmune manifestations via chronic inflammation (Reviewed in [183]) [184]. Thus, it is possible that while this HLA allele might protect from HIV infection, when it fails to do so, it could be deleterious to the individual.

Studies conducted with the Beninese CSWs cohort showed that while NK cells of HESN women did not cluster into a different subset when compared to uninfected women, they expressed higher levels of CD16 (FCɣRIII) [172]. CD16 is an Fc receptor that allows binding to IgG Fc portion, and whose binding is essential to ADCC activity by NK cells [165]. These findings suggest that NK cells from HESN women may have stronger ADCC capacities, which could be protective against HIV infection. ADCC was shown to correlate with the protection generated by the RV144 vaccination trial in Thailand, the only HIV vaccination trial that generated some form of protective immunity [185,186]. Moreover, elite controllers were found to have a stronger ADCC activity against HIV than normal progressors [187], and HIV-infected women who possessed CVL antibodies able to mediate ADCC were found to have a lower HIV RNA load in their genital tract [188]. As mentioned earlier, while no broadly neutralizing antibodies, nor Ig mediating ADCC activity were found in the blood or CVLs of Beninese HESNs (possibly due to lower levels and/or removal of the vaginal mucus prior to CVL sampling) [107], the possibility that other antibodies in their blood or mucosae could induce protective activity, notably IgG1 targeting gp41 and/or other antibodies produced by first-line B-cells such as the CD1c^+^ population [10], cannot be excluded. Besides, as mentioned earlier, ADCC is not the only protective mechanism that implies antibodies.

It is important to note that most of the studies presented above have been conducted with blood NK cells. Indeed, FGT NK cells differ from blood NK cells morphologically (the former are bigger and are more granular than the latter) and phenotypically (via the expression of CD69 and CD9, and levels of CD16 expression for example) [189,190]. These differences suggest that both subsets may possess different functions, and thus could contribute differently to HIV protection. Blood NK cells, notably the CD56^bright^CD16^−^ subset, are able to secrete the β-chemokines CCL3, CCL4 and CCL5 (RANTES), which are CCR5 ligands [191]. Interestingly, we have shown that Beninese HESNs harbor higher levels of these molecules in their blood [8]. High levels of these molecules could be protective against HIV via a competition with the virus for CCR5, one of the co-receptors necessary for viral entry [192]. Furthermore, uterine NK cells, mostly found in the FGT, were shown to inhibit HIV infection in vitro via the production of C-X-C Motif Chemokine Ligand (CXCL)12, a ligand for C-X-C chemokine receptor (CXCR)4, the other co-receptor used by HIV during infection [193]. While in vivo infections are almost exclusively done by CCR5-tropic (or R5) viruses, HIV-infected individuals harbor viruses tropic to both receptors, especially during late stages of infection (and even during treatment) [194]. Indeed, there is a phenotypic change between the Transmitted/Founder (T/F) virus and the myriad of quasispecies that are found in a viremic individual, with a tropism change from R5 to CXCR4-tropic (or X4), which correlates with progression to AIDS [194], although the mechanisms related to this phenotypic conversion remain to be elucidated. Nevertheless, viral resistance to CCR5 drugs such as Maraviroc is a testament of the importance of X4-tropic viruses in the infection and thus could reinforce the importance of uterine NK cells in inhibiting these types of viruses [195,196].

NK cells also seem to play a role in the rectal immunity to HIV. A higher frequency of NK cells in rectal mucosa (which resembles that of the FGT, with the lower expression of CD16 and a higher expression of CD56 compared to their blood counterparts) seem to correlate negatively with HIV-1 replication in this tissue, as opposed to MZ B-cells, which frequencies correlate positively with viral replication [190,197]. It was suggested that MZ could contribute to trans-infection of HIV given that as mentioned earlier, these cells can bind to virus particles via surface lectins such as α4β7 and DC-SIGN [152]. We have shown that innate CD1c^+^ B-cells in the FGT have the capacity of binding to HIV Env gp120 [10]. However, the true potential of trans-infection by these cells has yet to be elucidated.

Interestingly, IL-17A levels correlated strongly to p24 production in the rectal model. This is of importance, since we have shown that Beninese HESN women harbor lower levels of IL-17 in their FGT when compared to non-HESN women [11]. IL-17A levels likely reflect the activity of Th17 T-cells, prime targets for HIV, at mucosal infection sites [39,40,41]. Thus, the lower levels of IL-17 in the FGT of HESNs could reflect lesser availability of Th17 target cells and/or regulated activity.

## 6. Conclusions

**Figure 1:**
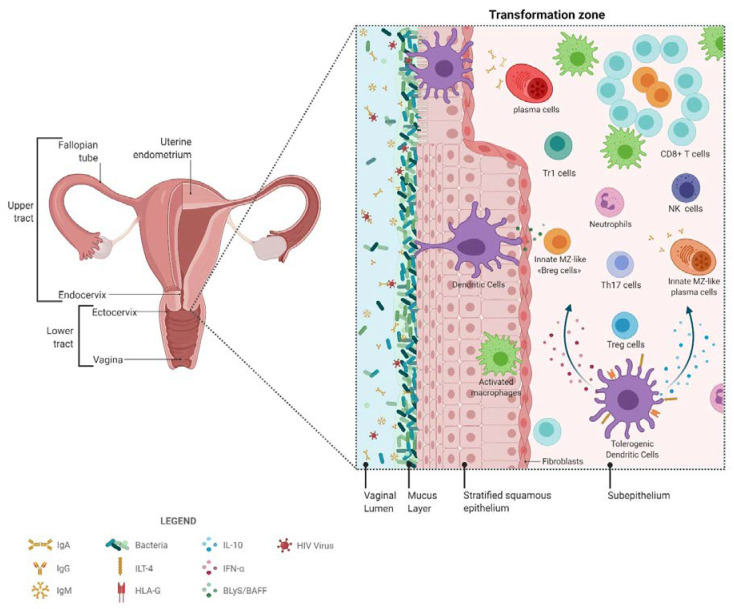
Active players in natural immunity to HIV.

Natural immunity to HIV in HESN CSWs, such as that found at the FGT mucosal portal of entry, likely involves a strong capacity to generate efficient anti-viral responses while simultaneously preventing excessive inflammation. This probably implicates orchestration of first-line innate immune responses in conjunction with matured high-affinity adaptive responses. As depicted in Figure 1, local regulatory populations such as “DC-10”-like, Treg, Tr1, and possibly CD1c^+^ “Bregs (Regulatory B-cells)” may help shape and maintain a low-inflammatory genital milieu, which allows for balanced responses from effector populations. It is easy to think that since expression and activity of NR4A molecules can be modulated therapeutically, such strategies may be contemplated in modulating the mucosal environment at the genital portal of entry to prevent HIV infection. Also, contained BAFF levels likely contribute to maintaining the integrity of innate CD1c^+^ “MZ-like” B-cells, which capacity of binding to HIV Env and producing IgG and/or IgA, raises particular interest because MZ B-cells can acquire Ig somatic mutations and could be harnessed to increase HIV Env affinity in the optics of designing preventive strategies involving the generation of protective first-line responses in the FGT.

The understanding of the genetic, environmental, and cellular mechanisms which undercover the potent anti-viral but regulated immune responses that prevent excessive immune activation and recruitment of HIV target cells at the initial site of exposure in the context of natural immunity are mandatory to the design of preventive devices against HIV infection.

## Data Availability

The data presented in this study are available on request from the corresponding author.

## Abbreviations

HESN	Highly Exposed Seronegative
CSW	Commercial Sex Worker
FGT	Female Genital Tract
MALT	Mucosal Associated Lymphoid Tissue
PRR	Pattern Recognition Receptor
TLR	Toll-like Receptor
CVL	Cervico-Vaginal Lavage
ADCC	antibody-dependent cellular cytotoxicity
ADCP	antibody-dependent cell phagocytosis
MoDC	Monocyte-Derived Dendritic Cell
Tregs	Regulatory T-cells
Tr1	T regulatory type 1
NR4A	Nuclear Receptors 4A
Bregs	Regulatory B-cells
BAFF	B-cell Activation Factor
MZ	Marginal Zone
iNKT	Invariant Natural Killer T-cells
KIR	Killer Immunoglobulin-like Receptors
TIGIT	T-cell Immunoreceptor with Ig and ITIM domains
